# Changes in Peripheral Blood Eosinophil Counts and Risk of Eosinophilic Granulomatosis with Polyangiitis Onset after Initiation of Dupilumab Administration in Adult Patients with Asthma

**DOI:** 10.3390/jcm12175721

**Published:** 2023-09-01

**Authors:** Yoshitomo Kushima, Yasuo Shimizu, Hiromi Hoshi, Ryo Arai, Naoya Ikeda, Yusuke Nakamura, Meitetsu Masawa, Hiroaki Okutomi, Nana Yazawa, Kazuyuki Chibana, Akihiro Takemasa, Seiji Niho

**Affiliations:** 1 Department of Pulmonary Medicine and Clinical Immunology, Dokkyo Medical University, Tochigi 321-0293, Japan; kushima@dokkyomed.ac.jp (Y.K.); hiromi-h@dokkyomed.ac.jp (H.H.); r-arai@dokkyomed.ac.jp (R.A.); nikeda@dokkyomed.ac.jp (N.I.); nakamuyu@dokkyomed.ac.jp (Y.N.); meitetsu@dokkyomed.ac.jp (M.M.); o-hiro@dokkyomed.ac.jp (H.O.); n-yazawa@dokkyomed.ac.jp (N.Y.); takemasa@dokkyomed.ac.jp (A.T.); siniho@dokkyomed.ac.jp (S.N.); 2 Department of Pulmonary Medicine, Dokkyo Medical University Nikko Medical Center, Nikko City 321-2335, Japan; kchibana@dokkyomed.ac.jp

**Keywords:** dupilumab, peripheral blood eosinophilia, eosinophilic granulomatosis with polyangiitis, asthma, rhinosinusitis

## Abstract

Background: The purpose of this study is to clarify the changes in peripheral blood eosinophil (PBE) counts and eosinophilic granulomatosis with polyangiitis (EGPA) onset in patients with asthma who were treated with dupilumab in clinical practice. Methods: The primary outcome of this study is to determine the onset of EGPA in patients whose PBE counts continued to rise within 6 months of dupilumab initiation (rising group) and in patients whose PBE counts peaked and subsequently declined within 6 months (peaked and declined group). As a secondary outcome, the incidence of developing EGPA in patients with PBE counts greater than 1500 cells/μL at 3 or 6 months after dupilumab administration is investigated. Results: A total of 37 individual were enrolled (male/female = 14/23, median age = 57.0 years old). The development of EGPA was significantly more frequent in the rising group compared with the peaked and declined group (*p* = 0.042, effect size = 0.455, moderate association). Patients with PBE counts greater than 1500 cells/μL showed a significantly higher risk of developing EGPA (*p* = 0.017, effect size = 0.678, strong association). Conclusions: Physicians should check for the onset of EGPA by monitoring the elevation of eosinophils within 6 months after dupilumab administration, especially in patients with PBE counts greater than 1500 cells/μL at 3 months.

## 1. Introduction

Dupilumab is a monoclonal antibody against human interleukin-4 receptor α (IL-4Rα), which broadly suppresses type 2 inflammation by inhibiting downstream signaling of IL-4 and IL-13 [1]. Asthma, atopic dermatitis, chronic rhinosinusitis with nasal polyps (CRwNP), and eosinophilic esophagitis are approved therapeutic indications for dupilumab, but the regulatory approval status in each country varies [2,3,4]. There are currently many clinical trials using dupilumab, and it is also likely that dupilumab will be used in the future in patients with a variety of diseases and in younger age groups [5].

The TRAVERS study evaluated the long-term safety and efficacy of dupilumab in asthma patients by combining data from those who completed multiple clinical trials of dupilumab, including EXPEDITION, DRI, QUEST, and VENTURE, and the study confirmed the long-term clinical benefit of dupilumab in improving asthma symptoms, maintaining long-term improvement in respiratory function, and reducing oral corticosteroids intake [6,7,8,9,10]. Although the clinical benefit of dupilumab has been confirmed, transient increases in peripheral blood eosinophilia are known to occur with dupilumab administration [10]. Furthermore, clinical practice has shown that blood eosinophilia in patients after dupilumab administration does not always uniformly decline after a transient rise [11]. In the TRAVERS study, 3.6% (93/2597) of the cases exhibited blood eosinophilia during treatment with dupilumab, including four cases of blood eosinophilia associated with notable clinical symptoms, eosinophilic granulomatosis with polyangiitis (EGPA), and lung infiltration [10]. EGPA is a systemic autoimmune disease in which an abnormal increase in eosinophils causes inflammation and injury in small- to medium-sized blood vessels, resulting in impaired blood flow, necrosis, and organ dysfunction, which requires early detection and treatment. The disease is unique because it combines asthmatic manifestations with hypereosinophilic disorders and ANCA-associated vasculitis features [12]. The TRAVERS study defined eosinophilia as a peripheral blood eosinophil (PBE) count greater than 3000 cells/μL [13]. A PBE count of 3000 cells/μL is 30–50% of the level that is considered normal for all leukocytes in adults, which is 5000–10,000 cells/μL [14]. Considering that the PBE count as a percentage of white blood cells in healthy individuals ranges from 1–4%, 30–50% is very high [15]. There is concern that using a PBE count of 3000 cells/µL as an indicator of eosinophilia in patients on dupilumab may delay the diagnosis of EGPA.

PBE counts can be affected by a variety of intrinsic and extrinsic factors [16,17], and some cytokines, such as vascular endothelial growth factor, affect the cell count in asthmatics [18,19]. Real-world data on dupilumab therapy for patients with asthma have shown that PBE counts and cardiac function should be monitored in patients with PBE counts ≥ 1500 cells/μL during dupilumab therapy [11]. Evidence is rising concerning possible surgical indications in patients developing EGPA after biologics therapy who need to suspend the monoclonal antibody [20,21]. It remains unknown whether paradoxical CRSwNP worsening during biological treatment could be an alarm for possible EGPA development [22,23]. In this context, it is important to clarify the relationship between changes in PBE counts induced by dupilumab and the onset of EGPA to ensure the safe use and benefits of dupilumab.

The purpose of this study is to retrospectively investigate and elucidate the changes in PBE count and EGPA onset in patients with moderate to severe asthma who were treated with dupilumab in clinical practice.

## 2. Materials and Methods

### 2.1. Study Design

This observational study involved the retrospective assessment of changes in the PBE counts and onset of EGPA after dupilumab administration in asthma patients. The surveillance period was designated from 1 April 2019 to 31 October 2022. Since PBE counts have typically returned to baseline or lower after the first 24 weeks (6 months) from dupilumab administration in patients with asthma and chronic rhinosinusitis with nasal polyp (CRSwNP) [13], 6 months after dupilumab administration was set as the data collection period for the primary outcome. Accordingly, the primary outcome of this study was to determine the onset of EGPA in patients whose PBE counts continued to rise within 6 months of dupilumab initiation (rising group) and in patients whose PBE counts peaked within 6 months and subsequently declined (peaked and declined group). Real-world data on dupilumab suggest that PBE counts should be monitored during dupilumab administration and cardiac function in patients with PBE counts greater than 1500 cells/μL [11]. Then, as a secondary outcome, patients with PBE counts greater than 1500 cells/μL at 3 or 6 months were compared with those with PBE counts less than 1500 cells/μL to determine whether any cases of EGPA onset were among them. 

Severity of asthma was determined based on the Global Initiative for Asthma [24], and moderate to severe asthma patients treated with dupilumab from 1 April 2019 to 31 October 2022 were included in the study. Inclusion criteria were established as follows: (i) patients who were treated at the Department of Pulmonary Medicine and Clinical Immunology, Dokkyo Medical University Hospital, with 600 mg of dupilumab by subcutaneous injection for the first treatment and 300 mg for the subsequent treatments every 2 weeks for at least 3 months according to manufacturer’s recommendation, (ii) changes in PBE counts could be observed for at least 3 months from the start of treatment, (iii) 16 years of age and older at the start of dupilumab administration. Exclusion criteria for the patients for the analysis were established as follows: (i) patients were excluded who had EGPA or a history of EGPA prior to dupilumab administration, (ii) PBE counts were never measured within the first 3 months of treatment, (iii) refusal to participate in the study. 

### 2.2. Data Collection

Data were extracted from digital medical records, including age, sex, body mass index (BMI), smoking status, and comorbidities. Prescriptions of pharmacotherapy were examined in regard to medications for asthma and allergies at the time of dupilumab initiation, as well as biologics prior to initiation. The frequency of asthma exacerbations was defined as the history of oral corticosteroid (OCS) bursts of more than 5 mg/day (prednisolone or other steroids converted to prednisolone) for longer than 3 consecutive days during the past 12 months before starting dupilumab. The frequency of hospitalizations due to asthma exacerbations during the past 12 months prior to the start of dupilumab was also surveyed. Pulmonary function tests (FUDAC-7 or FUDAC-77, Fukuda Denshi, Tokyo, Japan) and exhaled nitric oxide (FeNO) (NOA280i^®^, Sieverse Inc., Hamel, IL, USA, or NIOX VERO^®^ CHEST, Tokyo, Japan) values were taken from data obtained at the time of dupilumab initiation [25,26]. Levels of IgE in peripheral blood were only recorded at the start of dupilumab. PBE counts were recorded at the start of dupilumab and acquired every 3 months thereafter, with an allowance of ±1.5 months, and the data closest to the 3-month schedule were adopted. The diagnosis of EGPA was based on the guideline for the management of vascular syndrome JCS2017, referencing the Chapel Hill Consensus Conference Nomenclature of Vasculitides [27,28]. The study was conducted in accordance with the Declaration of Helsinki and approved by the Ethics Committee at Dokkyo Medical University Hospital (R-71-4J).

### 2.3. Safety Profile

The number of patients who experienced adverse events due to dupilumab administration during the study period was extracted from the digital medical records. Even if the same adverse event occurred more than once in one patient, the patient who had the adverse events was counted as one patient. If the same patient had several different adverse reactions, the number of patients was counted for each adverse reaction. Regarding the EGPA-related symptoms at the onset of EGPA, the following symptoms were investigated: wheezing; fever; weight loss; glove-sock-type paresthesia and motor disturbance due to poly-mononeuritis; gastrointestinal bleeding including abdominal pain and lower bleeding due to ischemic enteritis; purpura due to cutaneous vasculitis; polyarthritis; and myalgia [27,28].

### 2.4. Statistical Analysis

Data were presented as numbers and percentages (%) or median and interquartile ranges (IQR). PBE counts were used as descriptive statistics based on trends over time. The Mann–Whitney U test was used for continuous variables and Fisher’s exact test was used for categorical variables to compare differences between the PBE rising group and the peaked and declined group, as shown in Table A1. Fisher’s exact test and the effect size were used for comparing EGPA onset between the two groups. The effect size was calculated using Cramer’s V test. All statistical analyses with two-tailed tests less than 0.05 were considered significant. Statistical analyses were performed using SPSS Statistics 28 (IBM SPSS, Armonk, NY, USA). 

## 3. Results

**Figure 1:**
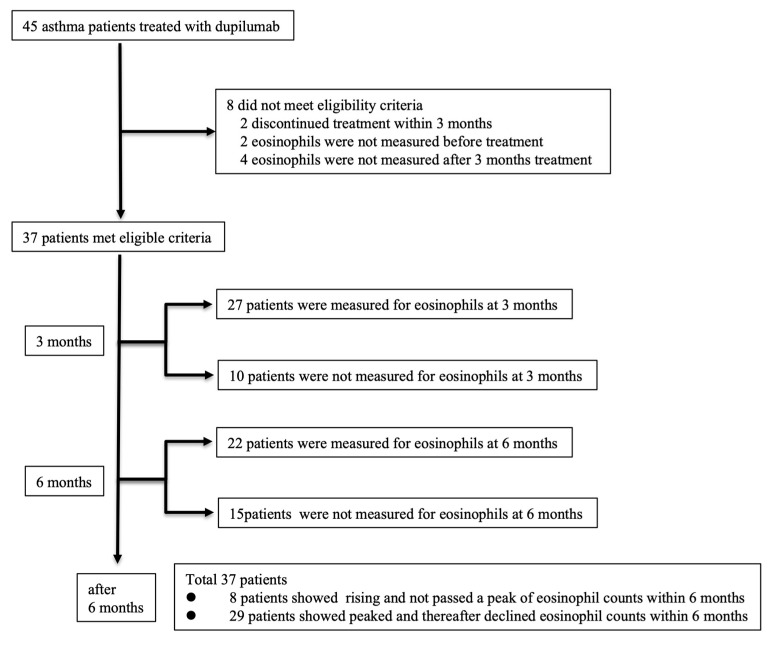
Enrollment and composition of patients included in the analysis.

Forty-five asthma patients received dupilumab between 1 April 2019 and 31 October 2022 (Figure 1). Among them, two patients discontinued dupilumab within 3 months, two did not have PBE measurements before dupilumab administration, and four did not have PBE measurements at any time after 3 months of dupilumab administration. These eight patients were excluded from the analysis based on the exclusion criteria. Finally, a total of 37 patients met the inclusion criteria for analysis. PBE counts were measured in 27 patients during the first 3 months of dupilumab treatment, and 10 patients were not measured within 3 ± 1.5 months. For these 10 patients, however, PBE counts were measured at 6 months or later. PBE counts were measured in 22 patients during the first 6 months of dupilumab treatment, and 15 patients were not measured within 6 ± 1.5 months. For these 15 patients, however, PBE counts were measured at 9 months or later. Finally, a total of 37 patients were included in the primary outcome analysis: the rising group included 8 patients, and the peaked and declined group included 29 patients. 

The patients’ backgrounds are shown in Table 1. The median age was 57 years, and 62.1% of the 37 patients were female. Furthermore, 48.6% of the patients had a smoking history (current or former), and 10.8% had a BMI > 30. Complications related to allergy and type 2 inflammation were as diverse as CRwNP, such that those related to smoking and metabolic diseases, specifically chronic obstructive pulmonary disease (COPD) and diabetes mellitus (diabetes), accounted for 10.8% and 13.5% of the patients, respectively. The mean daily dose of ICS was 640 μg/day, and the most commonly used inhaled therapy was triple therapy (ICS+LABA+LAMA). The proportion of patients taking leukotriene receptor antagonists was 89.1%. OCSs were taken by 43.2% of the patients, with a median dose of 4.5 mg/day. The use of biologics prior to dupilumab was observed in 51.3% of patients, mostly benralizumab and mepolizumab targeting IL-5/IL-5R. The proportion of patients with asthma exacerbations prior to dupilumab was 20.6%. Pulmonary function at the start of dupilumab treatment was measured in 64.9% of patients, with a low FEV/FVC(G) of 71.1%, and FeNO was observed at a high of 49.2 ppb. The median PBE count at the start of dupilumab treatment was relatively low, at 20 cells/μL.

**Figure 2:**
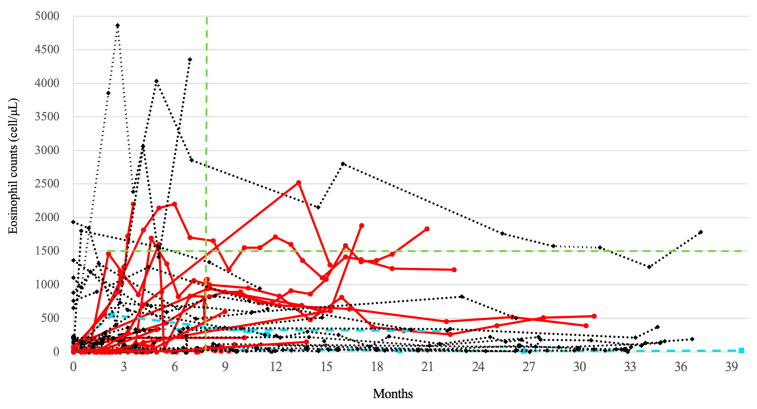
The overall trend in peripheral blood eosinophil (PBE) counts for patients after starting dupilumab therapy. PBE counts that could be tracked every 3 months over the follow-up period from 1 April 2019 to 31 October 2022 were indicated (*n* = 37). Black dotted lines are patients without premedicated biotherapy, red lines are patients premedicated with anti-IL-5/IL-5R antibody, and blue lines are patients premedicated with omalizumab. The horizontal green dotted line indicates a PBE count of 1500 cells/µL, and the vertical green dotted line indicates 6 months.

PBE counts were initially elevated after dupilumab treatment and then declined in most cases (Figure 2). Patients who had previously received anti-IL-5/IL-5R antibody therapy had low PBE counts at the start of dupilumab (red line in Figure 2). Regardless of whether biologics were premedicated or not, most patients showed a transient elevation of PBE after receiving dupilumab, but the PBE counts in some patients remained elevated (Figure 2). Comparing PBE counts of IL-5/IL-5R antibody users and non-users before dupilumab administration, baseline PBE was clearly higher in IL-5/IL-5R non-users (*p* < 0.001). At 3 months after dupilumab starting, the median [IQR] of PBE counts for IL-5/IL-5R non-users was 455 [35–1273] (cells/μL) and 40 [0–775] (cells/μL) for IL-5/IL-5R users, but there was no statistical difference. PBE counts at 6 months after dupilumab starting were not different between both groups (Table A3).

**Figure 3:**
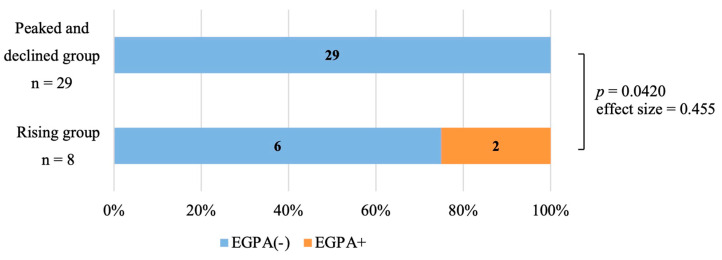
Onset of eosinophilic granulomatosis with polyangiitis (EGPA) in patients whose PBE counts continued to rise within 6 months of dupilumab initiation (rising group, *n* = 8) and in patients whose PBE counts peaked within 6 months and subsequently declined (peaked and declined group, *n* = 29). Two of the eight patients in the rising group had developed EGPA (orange box). Development of EGPA was significantly more frequent in the rising group compared with the peaked and declined group (*p* = 0.042, effect size = 0.455, indicating moderate association). Fisher’s exact test and the effect size were used to compare EGPA onset between the two groups. Effect size was calculated using Cramer’s V test.

Two of the patients in the rising group (2/8, 25%) developed EGPA, but there were no patients who developed EGPA in the peaked and declined group (0/29, 0%). Patients in the rising group were significantly more likely to develop EGPA compared with the patients in the peaked and declining group (*p* = 0.042, effect size = 0.455 indicating moderate association) (Figure 3). There were no significant differences in age, sex, baseline PBE count, or prior use of biologics between the two groups. Smoking status and comorbid allergies appeared to be more common in the rising group, but there was no significant difference (Table A1).

**Figure 4:**
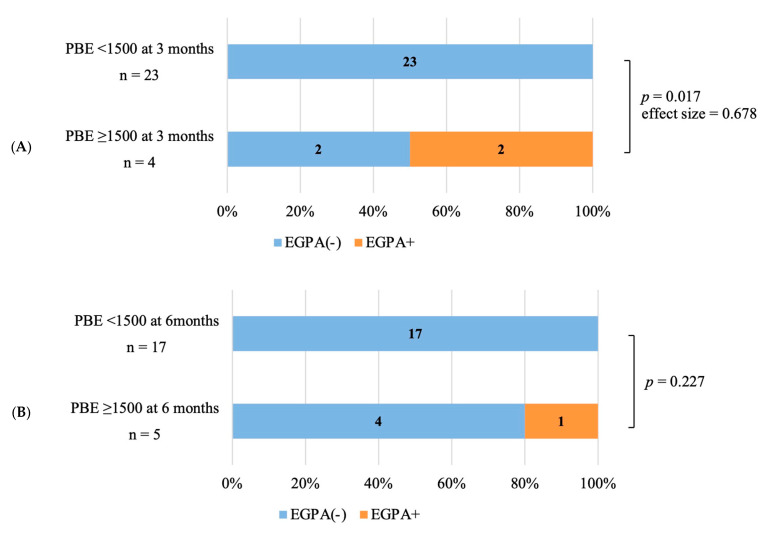
The incidence of developing EGPA in patients with PBE counts greater than 1500 cells/μL at 3 and 6 months after dupilumab administration. PBE counts were measured in 27 patients after 3 months of dupilumab administration. Among 27 patients, 4 patients showed ≥ 1500 cells/μL of PBE, and 23 patients showed <1500 cells/μL counts of PBE. Patients showing ≥ 1500 cells/μL of PBE counts included two patients who developed EGPA (2/4). A significant difference in the incidence of developing EGPA was observed between patients with PBE counts ≥ 1500 cells/μL at 3 months (2/4) and those with the lower counts (0/23) (*p* = 0.017, effect size of = 0.678) (**A**). PBE counts were measured in 22 patients after 6 months of dupilumab therapy. Among 22 patients, 5 patients showed ≥ 1500 cells/μL of PBE and 15 patients were <1500 cells/μL of eosinophils. Patients showing ≥ 1500 cells/μL of PBE included one patient who developed EGPA (1/5). A Significant difference in the incidence of developing EGPA was not observed between patients with PBE ≥ 1500 cells/μL at 6 months (1/5) and those with the lower counts (0/15) (**B**). Fisher’s exact test and the effect size were used to compare EGPA onset between the two groups. Effect size was calculated using Cramer’s V test.

**Figure 5:**
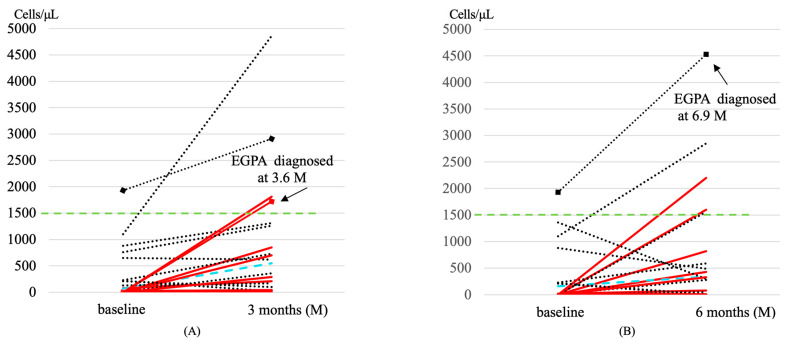
Changes in eosinophil counts of patients who underwent post-dupilumab therapy. PBE changes from the start of dupilumab treatment to 3 (**A**) and 6 months (**B**) are shown. The arrows indicate changes in PBE in patients who later developed EGPA. A patient diagnosed with EGPA at 3.6 months is shown in (**A**). This patient was not included in the observation of PBE changes at 6 months (**B**) because dupilumab was discontinued at the diagnosis of EGPA. Black dotted line: patients without biologics premedication, red line: patients premedicated with anti-IL-5/IL-5R, blue dotted line: patients premedicated with omalizumab. Green line: PBE counts 1500 cells/μL.

For the secondary outcome, the incidence of developing EGPA in the future for patients with PBE counts greater than 1500 cells/μL (2/4) was compared with those who had less than 1500 cells/μL (0/23) at 3 months after starting dupilumab treatment. Patients with PBE counts greater than 1500 cells/μL showed a significantly higher incidence of developing EGPA in the future compared with those who had less than 1500 cells/μL at 3 months (*p* = 0.017, effect size = 0.678 indicating strong association) (Figure 4A). The incidence of developing EGPA in the future for patients with PBE counts greater than 1500 cells/μL (1/5) was also compared with those who had less than 1500 cells/μL (0/17) at 6 months after starting dupilumab treatment. Patients with PBE counts greater than 1500 cells/μL did not show a significant difference in the incidence of developing EGPA in the future compared with those who had PBE counts less than 1500 cells/μL at 6 months (Figure 4B). The PBE counts were elevated in most of the patients during the first 3 months after starting dupilumab, and among these patients, those with PBE counts above 1500 cells/μL, including patients who developed EGPA later, showed a sharp increase in their PBE counts (Figure 5A). At 6 months after dupilumab initiation, the PBE counts were decreased in some patients, but those with PBE counts above 1500/μL, including a patient who developed EGPA later, showed a continued rise in their PBE counts (Figure 5B). For the patients diagnosed with EGPA at 3.6 or 6.9 months, dupilumab was discontinued at that point. The chief complaints reported at EGPA onset were fever and dyspnea in patients who developed EGPA at 3.6 months and dyspnea in patients who developed EGPA at 6.9 months.

The safety profile revealed that the most common adverse event related to dupilumab treatment was an injection-site reaction, and there were no serious adverse events (Table 2).

## 4. Discussion

This study focused on the elevated PBE counts in asthma patients and the onset of EGPA during dupilumab therapy. Asthmatics may occasionally have EGPA, and PBE is typically elevated in patients with EGPA. Treatment of asthmatic patients with dupilumab may also induce elevated PBE. This study suggests that PBE counts continue to rise within 6 months after dupilumab administration in patients who are at higher risk of developing EGPA, which should be considered during dupilumab therapy.

Several reports have previously examined changes in the PBE counts during dupilumab therapy. Dupin et al. examined 60 patients treated with dupilumab and found that the PBE counts did not necessarily rise transiently and mostly declined after dupilumab administration [11]. Comparing the backgrounds of the patients in Dupin’s study with those in our study, there were no remarkable differences in age, smoking status, comorbidities, or ongoing inhaler use for asthma therapy [11]. However, the daily dose of OCSs was lower and lung function was higher, and the baseline PBE count at the start of dupilumab treatment was lower in our study. Thus, the patients in our study had milder diseases than those observed in Dupin’s study [11]. This observation might be attributed to differences in prior biotherapy. In Dupin’s study, 83.9% of the 60 patients received omalizumab and 16.7% of patients received mepolizumab before administration of dupilumab, whereas 43% of the 37 patients received benralizumab and mepolizumab in our study, indicating that anti-IL-5 or anti-IL-5R was the most common pretreatment [11]. 

The impact of biotherapy on PBE counts after dupilumab administration has also been previously reported [29]. Numata et al. found that PBE counts peaked around 3 months in patients who had not received biologics prior to dupilumab but peaked around 6 months in patients who had received prior biologics, indicating a delayed peak PBE count [29]. As shown in Figure 4 and Table A3, we also observed that the PBE count appeared to decline at 6 months after dupilumab administration in patients who did not receive prior biologics, but this was not the case in patients who received prior biologics. Although the TRAVERSE study demonstrated that the PBE counts transiently rose and then fell after dupilumab therapy, this was not always the case, and some patients exhibited a different trend [10,11,29].

Regarding the relationship between developing EGPA and eosinophilia after dupilumab administration, Olaguibel JM et al. reviewed 61 cases prior to 19 March 2022 [30]. From our search on PubMed^®^ up until 31 May 2023, five additional cases of EGPA were reported in patients with severe asthma and nasal polyposis who were treated with dupilumab [31,32,33,34,35]. Among these five patients, one patient developed EGPA several months after the completion of dupilumab treatment [34], but other patients developed EGPA during dupilumab therapy [31,32,33,35].

By integrating the patients presented by Olaguibel JM et al. and these subsequent patients included in our study [30,31,32,35], 20 patients were evaluated regarding the PBE count and onset of EGPA during on-treatment after dupilumab administration. Of these 20 patients, 5 patients developed EGPA within 30 days after dupilumab therapy (peak range of eosinophil count: 400–10,280 μL), 9 patients developed EGPA between 30 and 180 days (peak range of eosinophil count: 2700–17,400 μL), and 6 patients developed EGPA after more than 180 days (peak range of eosinophil count: 7680–14,700 μL). Collectively, 14 of the 20 patients (70%) had developed EGPA within 180 days (6 months), and 6 of the 20 (30%) patients developed EGPA more than 180 days after dupilumab administration [30,31,32,35]. 

It has been unclear whether dupilumab administration is a trigger for the development of EGPA. Dupin et al. reported that there was no onset of EGPA in 60 dupilumab-treated asthmatic patients [11], and Numata et al. reported that 26 asthmatics on dupilumab did not develop EGPA, but rather, dupilumab was introduced as an asthma treatment in two patients with EGPA-complicated asthma [29]. However, since there have been case reports of dupilumab administration followed by the development of EGPA [30,31,32,33,34,35], the association cannot be ruled out. 

In the TRAVERSE study, asthmatic patients with PBE counts ≥1500 cells/µL at the time of administration were excluded. A PBE count of 1500 cells/μL represents a PBE count of 15–30% of the normal leukocyte count range [10,14]. High PBE counts are a risk factor for future asthma exacerbations, implying poor control; therefore, such patients are candidates for biologics therapy [36]. In addition, patients with high PBE levels are considered super responders of dupilumab [8]. Thus, patients with increased eosinophils may be candidates for dupilumab treatment. Since many clinical trials of dupilumab are currently underway and the expanding indication for dupilumab is expected in the future, it is necessary to consider the rising PBE counts and onset of EGPA after dupilumab administration when patients have asthma. 

It has been suggested that the mechanism of eosinophil upregulation by dupilumab treatment involves the blocking of IL-4 and IL-13 signals and enhancement of IL-5 signals, resulting in an upregulation of IL-5 and an increase in eosinophils [37]. Another hypothesis is that IL-4 signaling is inhibited by dupilumab, which leads to inhibition of the very-late-antigen-4 (VLA-4) vascular cell adhesion molecule-1 (VCAM-1) pathway and reduction in serum eotaxin-3 levels. Since these adhesion molecules and chemokines are required for the extravasation of eosinophils, the reduction in signaling leads to eosinophilia in the bloodstream, without eosinophils migrating to peripheral tissues. Despite the rise in circulative eosinophils, the supply of eosinophils from the bone marrow continues, which may increase the concentration of blood eosinophils further [8,30]. Moreover, elevated eosinophils in blood vessels may be activated and cause vasculitis by damaging the vascular endothelium [30]. Another hypothesis is that a patient may have EGPA intrinsically, but prior IL-5/IL-5R antibody therapy has suppressed the onset of symptoms, and the switch from these drugs to dupilumab may cause EGPA. Reduction in OCSs during dupilumab therapy may also result in the appearance of pre-existing EGPA. 

Most patients who developed EGPA during dupilumab therapy had severe asthma and/or nasal polyposis, and few patients had atopic dermatitis without asthma [13,30]. In patients with atopic dermatitis, conjunctivitis rather than EGPA has been reported during dupilumab therapy [38]. The reason for this difference remains unclear.

The most common adverse event observed in the present study was an injection-site reaction, but the frequency was similar to that reported in randomized control studies and clinical data [10,11]. Yet, eosinophil-driven events, such as eosinophilic pneumonia, erythema, fever, myalgia, arthralgia, myositis, radiculopathy, and atopic dermatitis, were reported [39]. These events were not found in the present study, but need careful medical examination.

The main limitation of this real-world study is that it was conducted retrospectively, and no minimum sample size was calculated. Furthermore, not all the subjects in the analysis had their blood drawn every 3 months. Thus, the study could not avoid selection bias and information bias.

Confounding factors for the risk of developing EGPA and patient backgrounds could not be statistically analyzed because of the absence of a corresponding group without dupilumab therapy, and characteristics of the patients might contribute to the onset of EGPA. Moreover, the number of patients with EGPA was too small to statistically analyze their clinical background. Differences between patients in the rising group and the peaked and declined group were compared based on their backgrounds, but there were no significant differences between the two groups. The possibility cannot be ruled out that patients who received prior administration of IL-5/IL-5R antibodies were originally selected because of their high PBE counts. The involvement of selection bias/confounding bias in dupilumab administration cannot be ruled out. A prospective study with a larger patient population is needed to examine the outcomes of dupilumab treatment.

## 5. Conclusions

Dupilumab is a highly effective agent in moderate to severe asthma, but for optimal use, it is recommended that physicians check for the onset of EGPA by noting the rising PBE count during the first 6 months after dupilumab administration. Additionally, it may be useful to diagnose the presence or absence of EGPA complications prior to dupilumab administration.

## Figures and Tables

**Table 1:** Background of patients with moderate to severe asthma at the start of dupilumab treatment.

		*n* ^a^	Median [IQR] or *n* (%)
Demographics	Age (y), median [IQR]	37	57.0 [49–70.5]
	Sex: female, *n* (%)	37	23 (62.1)
	BMI (Kg/m^2^), median [IQR]	37	25.8 [22.8–28.8]
	Obesity (BMI > 30) *n* (%)	37	4 (10.8)
	Current smokers, *n* (%)	37	2 (5.4)
	Former smokers, *n* (%)	37	16 (43.2)
Comorbidities	CRwNP, *n* (%)	37	10 (27.0)
	Pollinosis, *n* (%)	37	5 (13.5)
	Allergic rhinitis, *n* (%)	37	5 (13.5)
	Diabetes, *n* (%)	37	5 (13.5)
	Chronic sinusitis, *n* (%)	37	4 (10.8)
	COPD, *n* (%)	37	4 (10.8)
	Food allergy, *n* (%)	37	4 (10.8)
	Eosinophilic otitis media, *n* (%)	37	3 (8.1)
	Atopic dermatitis, *n* (%)	37	3 (8.1)
	ABPA, *n* (%)	37	2 (5.4)
	AERD, *n* (%)	37	1 (2.7)
	CEP, *n* (%)	37	1 (2.7)
	Eosinophilic gastroenteritis, (%)	37	1 (2.7)
Ongoing treatments *	ICS+LABA+LAMA, *n* (%)	37	22 (59.5)
	ICS+LABA, *n* (%)	37	13 (35.1)
	ICS+LAMA, *n* (%)	37	1 (2.7)
	ICS, *n* (%)	37	1 (2.7)
	Dose of ICS (μg/d), median [IQR]	37	640 [320–800]
	LTRA, *n* (%)	37	33 (89.1)
	Macrolide, *n* (%)	37	9 (24.3)
	Theophylline, *n* (%)	37	9 (24.3)
	Daily OCS, yes, *n* (%)	37	16 (43.2)
	Dose of OCS (mg/d), median [IQR]	16	4.5 [2.6–5.0]
Previous biologics	Biologics, yes, *n* (%)	37	19 (51.3)
	Benralizumab, *n* (%)	37	11 (29.7)
	Mepolizumab, *n* (%)	37	5 (13.5)
	Omalizumab, *n* (%)	37	3 (8.1)
Asthma control	Exacerbations during the 12 previous months, *n* (%)	34	7 (20.6)
	Hospitalization in the past 12 months, *n* (%)	34	2(5.8)
Pulmonary function tests	FEV1 (mL), median [IQR]	25	2030 [1505–2525]
	FEV1 (% predicted)	25	86.1 [67.6–93.5]
	FEV1/FVC (%), median [IQR]	25	71.1 [60.0–79.4]
	FeNO (ppb), median [IQR]	33	49.2 [23.3–95.0]
Blood test	Total IgE level (kU/L), median [IQR]	34	123.3 [53.5–527.0]
	PBE count at baseline (/mm^3^), median [IQR]	37	20 [0–210]
	PBE count at baseline ≥ 150/mm^3^, *n* (%)	37	11 (29.7)
	PBE count at baseline ≥ 300/mm^3^, *n* (%)	37	6 (16.2)
	PBE count at baseline ≥ 1500/mm^3^, *n* (%)	37	1 (2.7)

A total of 37 individuals met the inclusion criteria for analysis (*n* = 37). Abbreviations: PBE, peripheral blood of eosinophils; CRwNP, chronic rhinosinusitis with nasal polyp; ABPA, allergic bronchopulmonary aspergillosis; AERD, aspirin-exacerbated respiratory disease; CEP, chronic eosinophilic pneumonia; ICS, inhaled corticosteroids; LABA, long-acting beta-agonist; LAMA, long-acting muscarinic agonist; FEV1, forced expiratory volume in 1 s; FVC, forced vital capacity; FeNO, fraction of exhaled nitric oxide. The *n* in the column indicates the number of patients, and (%) indicates the proportion of the number of patients. ^a^ Missing data were excluded. * The names of the drugs used in ICS, LABA, LAMA, and OCS are indicated in Table A2.

**Table 2:** Number of patients with each adverse event treated with dupilumab during the survey.

Adverse Events	Number of Patients (%)
Injection-site reaction	6 (16.2)
Pruritus	3 (8.1)
Drowsiness	2 (5.4)
Headache	1 (2.7)
Sputum	1 (2.7)
Nausea	1 (2.7)
Diarrhea	1 (2.7)
Joint pain	1 (2.7)

Even if an adverse event occurred more than once in one patient, the patient who had the side effect was counted as one patient. If the same patient had several different adverse events, they were also counted as duplicates in the number of patients experiencing each side effect. The *n* in the column indicates the number of patients with adverse drug reactions, and the (%) indicates the percentage of patients with adverse drug reactions relative to the total number of patients treated with dupilumab (*n* = 37).

## Data Availability

The data that support the findings of this study are available from the corresponding author upon reasonable request.

## Appendix A

**Table A1:** Background of patients whose peripheral blood eosinophil (PBE) counts peaked within 6 months and subsequently declined and patients whose PBE counts continued to rise within 6 months of initiation of dupilumab.

		Peaked and Declined Group		Rising Group		*p* Value
		*n* ^a^	Median [IQR] or *n* (%)	*n* ^a^	Median [IQR] or *n* (%)	
Demographics	Age (y), median [IQR]	29	57.0 [49–70.5]	8	51.0 [45.3–70.5]	0.388
	Sex: female, *n* (%)	29	17 (58.6)	8	6 (72.5)	0.683
	BMI (Kg/m^2^), median [IQR]	29	25.8 [22.6–28.6]	8	26.6 [24.6–29.9]	0.479
	Obesity (BMI > 30) *n* (%)	29	2 (6.9)	8	2 (25.0)	0.198
	Current smokers, *n* (%)	29	2 (6.9)	8	0 (0)	1.000
	Former smokers, *n* (%)	29	14 (48.2)	8	2 (25.0)	0.423
Comorbidities	CRSwNP, *n* (%)	29	7 (24.1)	8	3 (37.5)	0.665
	Pollinosis, *n* (%)	29	6 (20.7)	8	0 (0)	0.305
	Allergic rhinitis, *n* (%)	29	5 (17.2)	8	0 (0)	0.564
	Diabetes, *n* (%)	29	5 (17.2)	8	0 (0)	0.564
	Chronic sinusitis, *n* (%)	29	2 (6.9)	8	2 (25.0)	0.198
	COPD, *n* (%)	29	4 (13.8)	8	0 (0)	0.557
	Food allergy, *n* (%)	29	4 (13.8)	8	0 (0)	0.557
	Eosinophilic otitis media, *n* (%)	29	1 (3.4)	8	2 (25.0)	0.112
	Atopic dermatitis, *n* (%)	29	3 (10.3)	8	0 (0)	1.000
	ABPA, *n* (%)	29	2 (6.9)	8	0 (0)	1.000
	AERD, *n* (%)	29	1 (3.4)	8	0 (0)	1.000
	CEP, *n* (%)	29	1 (3.4)	8	0 (0)	1.000
	Eosinophilic gastroenteritis, (%)	29	1 (3.4)	8	0 (0)	1.000
Ongoing treatments	ICS+LABA+LAMA, *n* (%)	29	17 (58.6)	8	5 (62.5)	1.000
	ICS+LABA, *n* (%)	29	10 (34.5)	8	3 (37.5)	1.000
	ICS+LAMA, *n* (%)	29	1 (3.4)	8	0 (0)	1.000
	ICS, *n* (%)	29	1 (3.4)	8	0 (0)	1.000
	Dose of ICS (μg/d), median [IQR]	29	640 [320–800]	8	535 [170–800]	0.429
	LTRA, *n* (%)	29	25 (64.1)	8	8 (100)	0.557
	Macrolide, *n* (%)	29	5 (17.2)	8	4 (50.0)	0.078
	Theophylline, *n* (%)	29	9 (31.0)	8	0 (0)	0.169
	Daily OCS, yes, *n* (%)	29	12 (41.3)	8	4 (50.0)	0.705
	Dose of OCS (mg/d), median [IQR]	12	5.0 [3.0–5.0]	4	2.8 [2.1–10.1]	0.520
Previous biologics	Biologics, yes, *n* (%)	29	13 (44.8)	8	6 (75.0)	0.480
	Benralizumab, *n* (%)	29	6 (20.7)	8	5 (62.5)	0.035
	Mepolizumab, *n* (%)	29	4 (13.8)	8	1 (12.5)	1.000
	Omalizumab, *n* (%)	29	3 (10.3)	8	0 (0)	1.000
Asthma control	Exacerbations during the 12 previous months, *n* (%)	26	4 (15.4)	8	3 (37.5)	0.315
	Hospitalization in the past 12 months, *n* (%)	26	1 (3.4)	8	1(12.5)	0.421
Pulmonary function tests	FEV1 (mL), median [IQR]	20	1985 [1483–2230]	5	2630 [1705–3220]	0.110
	FEV1 (% predicted)	20	84.1 [60.8–92.3]	5	107.6 [77.1–112.6]	0.127
	FEV1/FVC (%), median [IQR]	20	70.1 [59.5–79.6]	5	77.9 [67.5–81.4]	0.267
	FeNO (ppb), median [IQR]	26	42.5 [20.5–94.0]	7	57.0 [49.2–108.0]	0.199
Blood test	Total IgE level (kU/L), median [IQR]	27	116.4 [37.5–745.9]	7	130.1 [79.9–454.0]	0.559
	PBE at baseline (/mm^3^), median [IQR]	29	20 [0–210]	8	0.0 [0.0–195.0]	0.483
	PBE at baseline ≥ 150/mm^3^, *n* (%)	29	9 (31.0)	8	2 (25.0)	1.000
	PBE at baseline ≥ 300/mm^3^, *n* (%)	29	5 (17.2)	8	1 (12.5)	1.000
	PBE at baseline ≥ 1500/mm^3^, *n* (%)	29	0 (0)	8	1 (12.5)	0.216

Comparing the backgrounds of the two groups (i.e., patients whose PBE counts peaked within 6 months and subsequently declined (peaked and declined group, *n* = 29) and patients whose peripheral blood eosinophil counts continued to rise within 6 months of initiation of dupilumab (rising group, *n* = 8)), prior administration of benralizumab was more common in the rising group (*p* < 0.05). Refer to Table 1 for abbreviations. ^a^: Missing data were excluded.

**Table A2:** Ongoing inhaled therapy and OCS at the start of dupilumab treatment (*n* = 37).

	Ongoing Treatments	Number of Patients (%)
ICS+LABA+LAMA	BUD/FORM+TIO	5 (13.5)
	FP/FORM+TIO	5 (13.5)
	FF/VI+TIO	4 (10.8)
	FF/VI/UMEC	3 (8.1)
	BUD/FORM/GLY	2 (5.4)
	MF/IND/GLY	1 (2.7)
	FF/VI+GLY	1 (2.7))
	BUD/FORM+UMEC	1 (2.7)
ICS+LABA	FF/VI	7 (18.9)
	BUD/FORM	4 (10.8)
	FP/FORM	2 (5.4)
ICS+LAMA	CIC+TIO	1 (2.7)
ICS	CIC	1 (2.7)
OCS	Prednisone	14 (37.8)
	Dexamethasone	2 (5.4)

The *n* in the column indicates the number of patients, and the (%) indicates the percentage of patients with drugs relative to the total number of patients treated with dupilumab (*n* = 37). BUD, budesonide; FP, fluticasone propionate; FF, fluticasone furoate; MF, mometasone furoate; CIC, ciclesonide; FORM, formoterol; VI, vilanterol; IND, indacaterol; TIO, tiotropium; UMEC, umeclidinium; GLY, glycopyrronium.

**Table A3:** The comparison of PBE counts after initiation of dupilumab with or without previous anti-IL-5/IL-5R therapy.

	Not Treated by Anti–IL–5/IL–5R		Treated by Anti–IL–5/IL–5R		*p* Value
	*n* ^a^	Median [IQR]	*n* ^a^	Median [IQR]	
PBE at baseline (/mm^3^), median [IQR]	21	160 [10–705]	16	0 [0–15]	<0.001
PBE at 3 months (/mm^3^), median [IQR]	14	455 [35–1273]	13	40 [0–775]	0.237
PBE at 6 months (/mm^3^), median [IQR]	13	300 [20–1075]	9	330 [5–1210]	0.844

The proportion of patients who had used anti-IL-5/IL-5R prior to dupilumab administration was 43.2%. ^a^: Missing data were excluded.
